# Patent Omphalomesenteric Duct with Protruding Bowels through a Ruptured Omphalocele

**DOI:** 10.4274/balkanmedj.2017.0230

**Published:** 2018-02-20

**Authors:** Emil Mammadov

**Affiliations:** 1 Department of Pediatric Surgery, Near East University School of Medicine, Nicosia, Cyprus

Persistent omphalomesenteric duct is a rare congenital anomaly and protrusion of the bowel through the duct is the only neonatal emergent clinical entity associated with this condition. The pathogenesis is the lack of intestinal involution by the ninth gestational week. There are several associated anomalies like intestinal malrotation, oesophageal atresia and omphalocele, which are all extremely rare (1,2,3,4,5). Herein, we present our case of successful surgical correction of this anomaly.

A male baby was delivered by C/S in the 32^nd^ gestational week due to foetal distress. Upon newborn examination, a bowel segment protruding from the caudal portion of a small omphalocele was detected. The distal part of the bowel seemed to be in a state of vascular compromise (red arrow, Figure 1). The anomaly initially looked like a ruptured omphalocele. Total blood count, biochemistry and abdominal ultrasonography were normal. Pulmonary hypertension was detected by echocardiography. Informed consent was obtained from the family. Emergent exploration was performed through the caudal portion of the omphalocele. Patent omphalomesenteric duct (yellow arrow, Figure 2) with prolapse of both proximal and distal segments of adjacent terminal ileum were noted. The bowel was reduced; resection of the patent omphalomesenteric duct and adjacent bowel with anastomosis and umbilicoplasty (Figure 3) was performed. Bowel exploration showed no additional gastrointestinal pathology. Enteral feeding was initiated on the second postoperative day and the surgical course was uneventful. After completion of the treatment for pulmonary hypertension, the baby was discharged from the hospital on the 15^th^ day. Pathologic examination of the excised specimen showed eroded and congested columnar epithelium without any sign of ectopic mucosa.

## Figures and Tables

**Figure 1:**
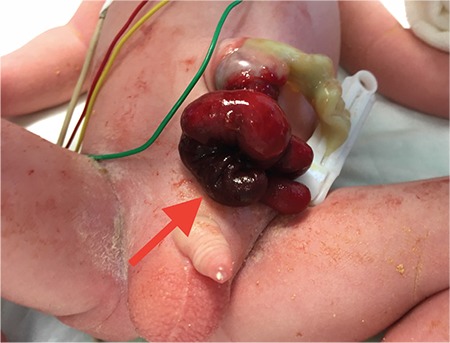
Bowel segments protruding from caudal portion of a small omphalocele. Distal part of the protruded bowel is compromised (red arrow).

**Figure 2:**
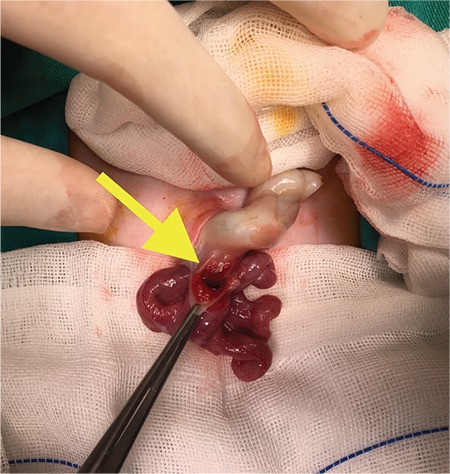
Patent omphalomesenteric duct (yellow arrow) upon exploration.

**Figure 3:**
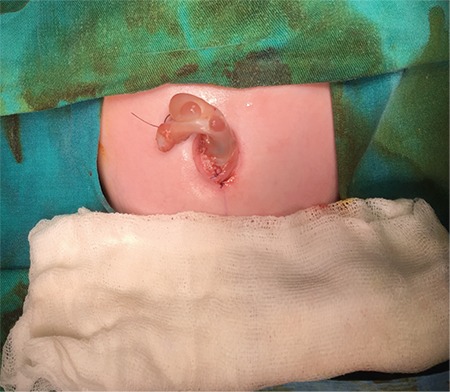
Completed umbilicoplasty view.
